# Supplementary figures and images for: Testing the validity and adequacy of linguistic phylogenetic analyses
Source: PLoS Comput Biol. 2026 May 20;22(5):e1014312. doi: 10.1371/journal.pcbi.1014312 (PMC13218487; doi:10.1371/journal.pcbi.1014312)

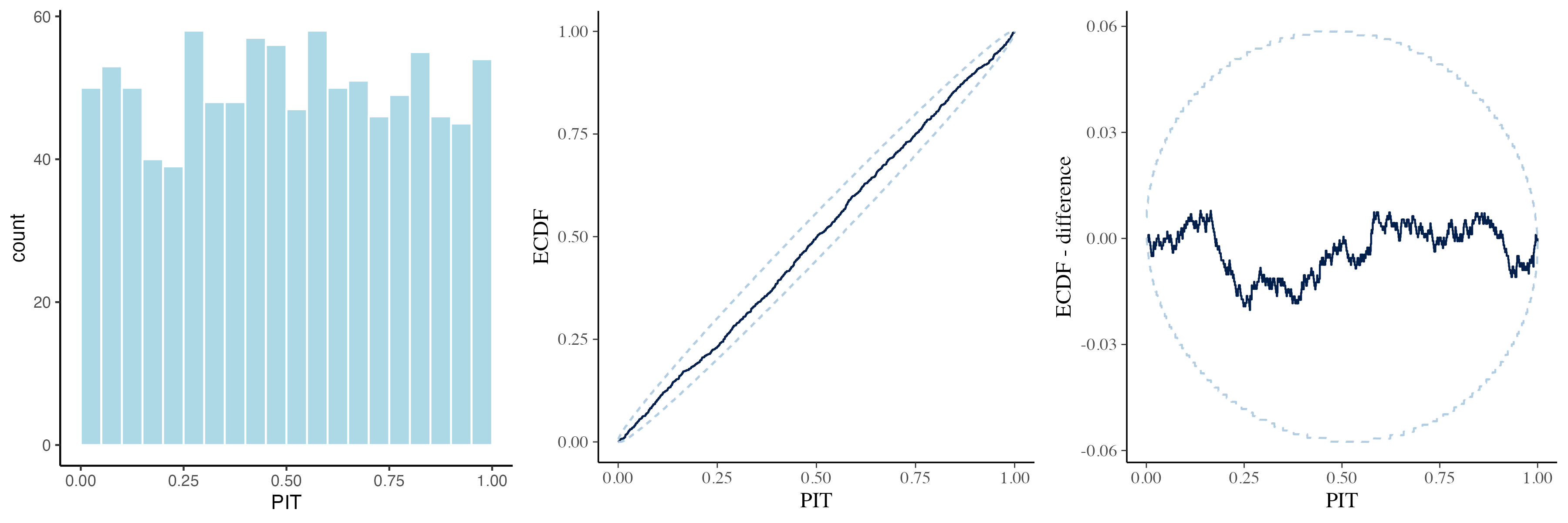

Supplement: S1 Fig — (JPG) [file pcbi.1014312.s001.jpg]

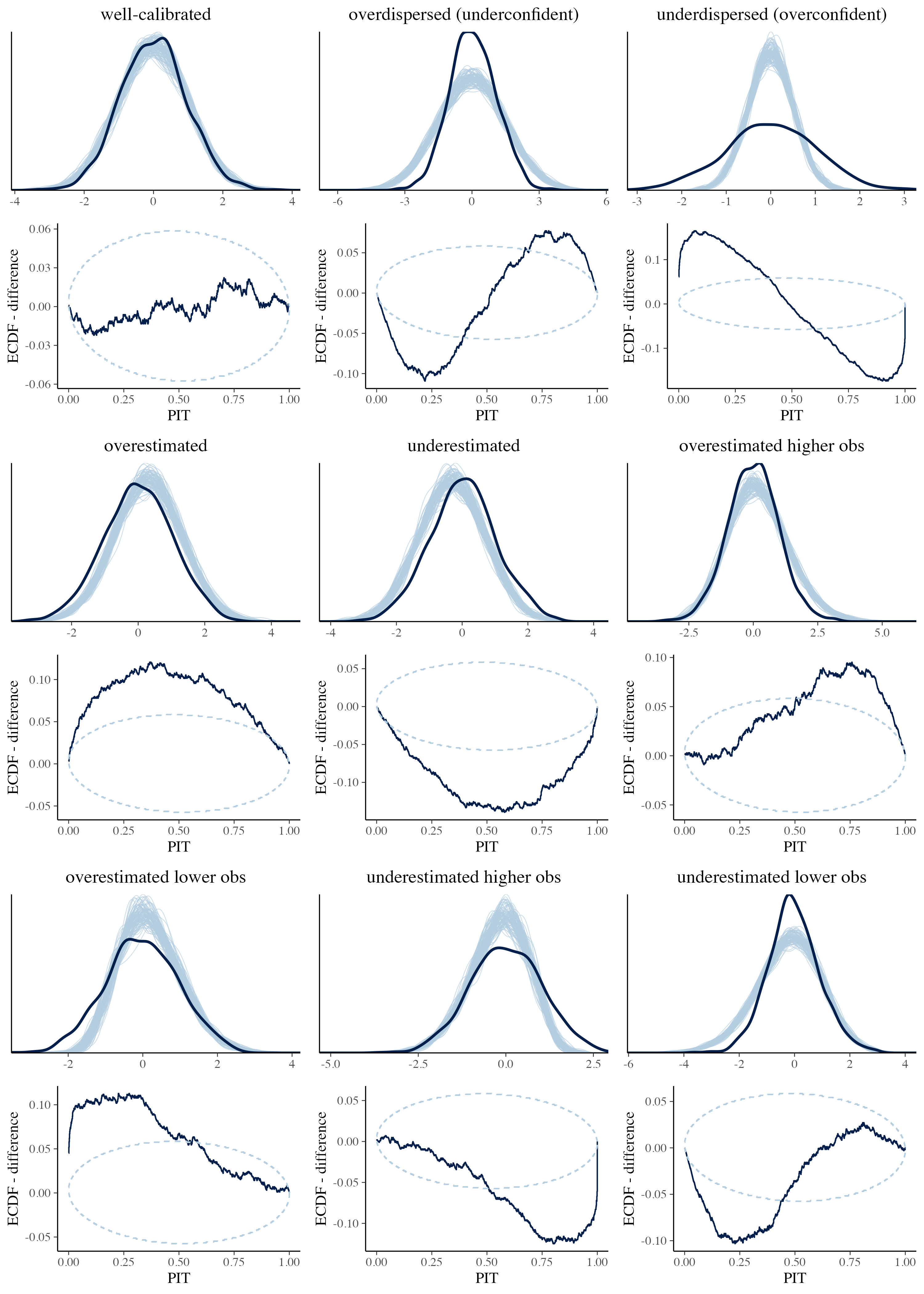

Supplement: S2 Fig — (JPG) [file pcbi.1014312.s002.jpg]

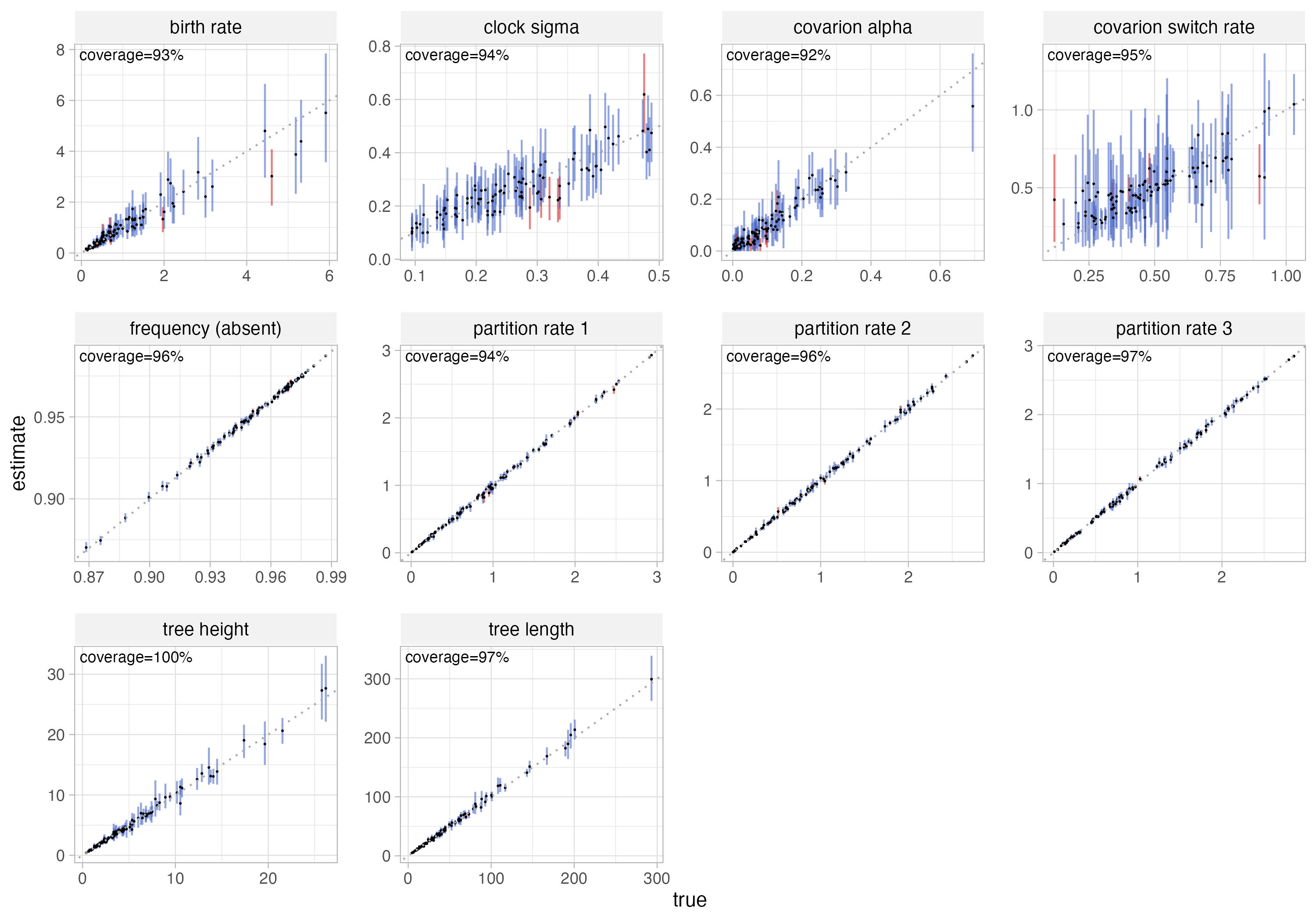

Supplement: S3 Fig — Ascertainment cognates are not removed in this implementation. (JPG) [file pcbi.1014312.s003.jpg]

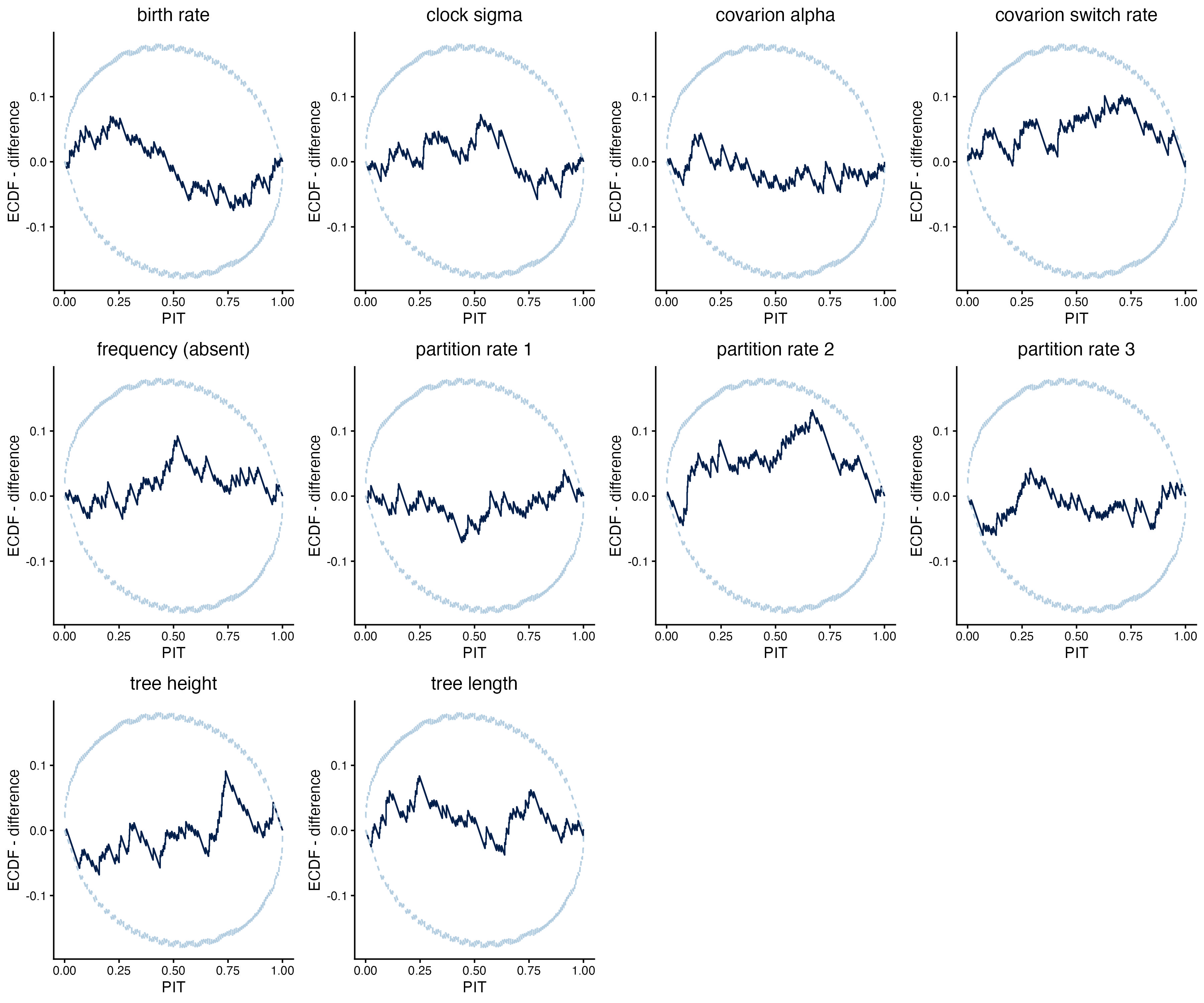

Supplement: S4 Fig — Ascertainment cognates are not removed in this implementation. (JPG) [file pcbi.1014312.s004.jpg]

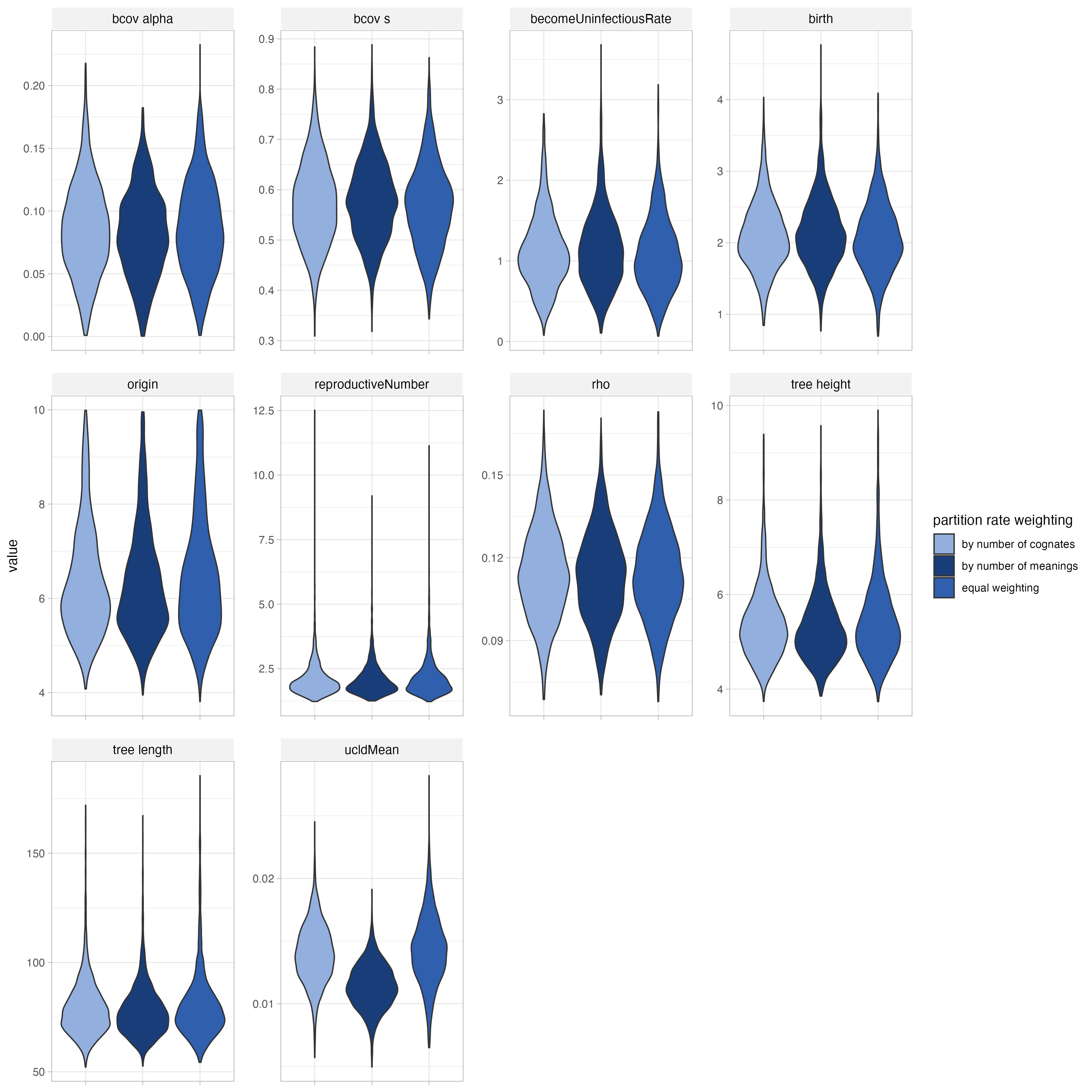

Supplement: S5 Fig — From an analysis on Indo-Iranic languages. (JPG) [file pcbi.1014312.s005.jpg]

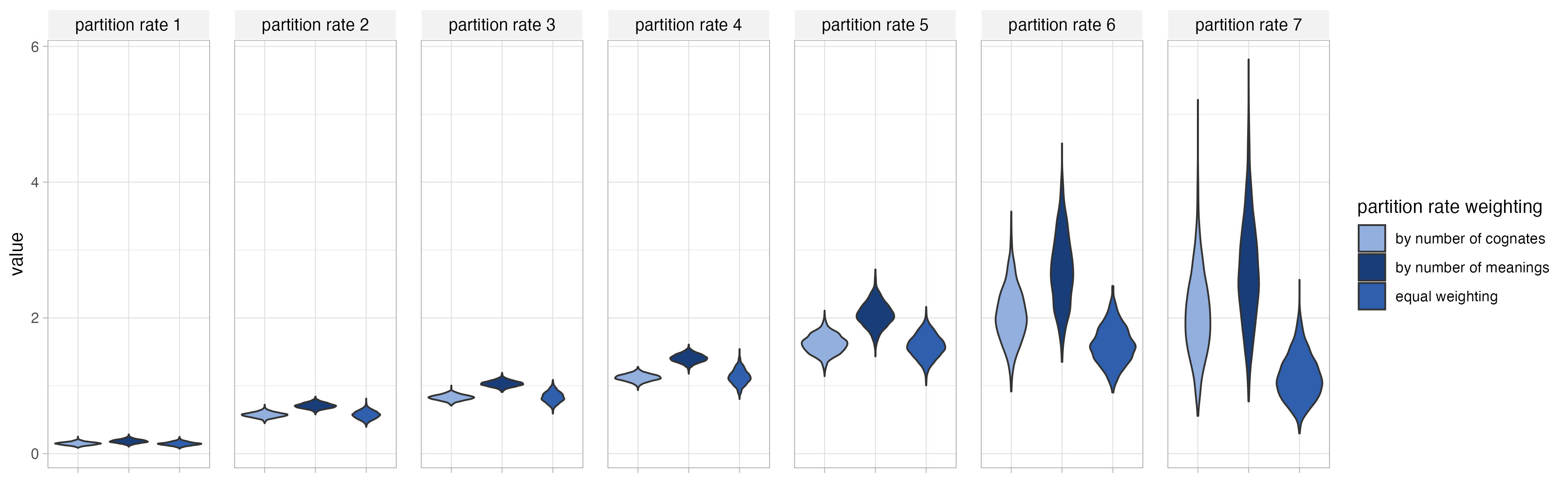

Supplement: S6 Fig — From an analysis on Indo-Iranic languages. (JPG) [file pcbi.1014312.s006.jpg]
