# Supplementary material for: Testing the validity and adequacy of linguistic phylogenetic analyses
Source: PLoS Comput Biol. 2026 May 20;22(5):e1014312. doi: 10.1371/journal.pcbi.1014312 (PMC13218487; doi:10.1371/journal.pcbi.1014312)
Supplement: Attachment — Submitted filename: review_PLOS.pdf [file pcbi.1014312.s007.pdf]

Review of ”Testing the validity and adequacy of linguistic phylogenetic analyses”

March 10, 2026

The author proposes a statistical test of the standard phylolinguistics framework, using SBC (Simulation Based Calibration). This topic is timely and well suited to PLOS Computational Biology. I believe it will be of interest to the scientific community, and hope it will eventually be published.

For context, I am a statistician with long experience in phylolinguistics. Overall, I found the article to be well written. I have a number of comments and requests for clarification. My assumption is that these will relatively straightforward to implement. I am nonetheless recommending a major revision because of point 1 below.

1 Major comments

1. The code and data should be made available on a public repository such as github. I would have liked to check the code and reproduce the figures, but was unable to do so. When the paper is revised, I would like to be able to verify the reproducibility.
2. The paper discusses ascertainment bias and partition rate re-weighting. It would be a good idea to explain these two notions in more detail, including with some technical discussion of they are implemented. In fact, to the uninformed reader, it might not be clear whether rate re-weighting is a technique for ascertainment bias or is a separate issue.
3. Linked to the previous comment: in the description of the 6 models, specify what was done for ascertainment bias correction.
4. There is no discussion of checking the convergence and mixing of the MCMC algorithms.
5. I am confused by the claim line 167 that rate parameters, tree height, and tree length are well-calibrated. For these quantities to be well estimated, we typically need some constraints on internal node ages, but these constraints were never

mentioned. (Without constraints, you can get the same likelihood by multiplying all branch lengths by a constant, and multiplying the rates by the same constant).

6. Various metrics are designed to search for mis-specification, but they are never described, nor even listed apart from the labels of Figure 6. Please describe these and give intuition of why they are interesting quantities.

7. Various mis-specifications are listed at the end of the final section. But do these mis-specifications matter? Do we have reason to believe that missing these mis-specifications lead to poor inference of the quantities of interest?

2 Minor comments

8. line 47, the term "rates of lexical replacement" is ambiguous: it is easy to interpret it as the rate at which a cognacy class changes in the multistate model. I would suggest the more neutral "transition rate".

9. If I understand correctly line 105, the simulated data sets comprise close to 30 000 cognacy classes. This is about 10 times larger than typical analyses in phylolinguistics, and should be commented upon.

10. In Figure 1, I don't understand what "$i \in 3$" and "$i \in 58$" mean.

11. I got confused by the numbering of the studies: when you refer e.g. to "analysis 3", it isn't clear if this is synthetic data analysis 3 or empirical data analysis 3. Maybe rename the analyses.

12. p. 5: note that if there were missing data, that would change the ascertainment: in practice, data which are absent or missing at all leaves are removed (even though the cognate may have in fact been present at a leaf where it is recorded as missing). This is discussed e.g. by Ryder and Nicholls (2011), for the less-used Stochastic Dollo model.

13. The interpretation of the SBC plots could go into more detail. For instance, remind us how to interpret the profiles of the plots you obtain.

14. Table 2: this is the *log*-marginal likelihood. Also, these are Bayes factors *against* each model. At first glance, I interpreted the 85.7 BF as meaning that the first line was the favoured model.

15. I don't understand the statement l. 215 that all cognate sets are eventually lost. Do you mean that they eventually become impossible to identify because of phonological changes? That is a separate issue.
