# Supplementary material for: Testing the validity and adequacy of linguistic phylogenetic analyses
Source: PLoS Comput Biol. 2026 May 20;22(5):e1014312. doi: 10.1371/journal.pcbi.1014312 (PMC13218487; doi:10.1371/journal.pcbi.1014312)
Supplement: Attachment — Submitted filename: Response to reviewers.pdf [file pcbi.1014312.s009.pdf]

Additional Academic Editor Comments:

King has provided an important and much needed study on characterising the statistical properties of the widely-applied, but not well-understood, binary covarion model used in phylogenetic linguistics. The author has confirmed that the standard BEAST 2 implementation is indeed well-calibrated (using the standard methodologies used to validate most Bayesian phylogenetic models), but it is only valid under a certain set of conditions. The author also tested the adequacy of this method at explaining real data, finding that it does not capture some of the subtleties found in empirical datasets.

The paper is well-written and was met with overall positive feedback from three expert Reviewers. However, there are some major changes required before this manuscript can be recommended for publication.

Please note that there are 3 Reviewers, with the comments of Reviewer 3 uploaded separately as a PDF.

My own comments are below, coming from the perspective of a software developer for BEAST 2. In particular, I am concerned about the novelty behind some aspects of this work.

The reported issue behind partition weighting was already picked up by the core BEAST 2 team in mid 2025. The fix was released as part of BEAST 2.7.8 on June 18 2025. I am unsure what BEAST 2 version is being used in the present study. This issue behind Dirichlet priors and the weighted delta exchange operator has been around for a long time, and I believe it can even be traced back to before the BEAST/BEAST2 split. The relevant information on the fix is below:

BEAST 2.7.8 patch nodes:
https://www.beast2.org/2025/06/29/what-is-new-in-v2.7.8.html
GitHub issues:
https://github.com/BEAST2-Dev/BEASTLabs/issues/22
https://github.com/CompEvol/beast2/issues/1199

The experiments conducted here by the developers have not been published in any peer-reviewed journal setting, but there are simulation studies above similar to the ones conducted here by the author.

This aspect of the findings made in the present study therefore does not appear to be novel, in which case it would be a deduction against the overall level of contribution made by this study. The author should acknowledge these recent changes made to the BEAST 2 core, noting whether their own experiments were conducted before or after the fix, run new experiments where appropriate, and reevaluate the conclusions of the manuscript accordingly.

All analyses were run in version beast2.7.8, so the issues described here are separate from the pre-2.7.8 bugs. The problems described in the paper are about the way the analysis is set up in the xml, rather than bugs in the beast2 code base.

Minor issues

Line 44: I am not sure what beta refers to in this equation, and I cannot find any other uses of this variable in the manuscript.

Beta is the normalising constant. For simplicity I removed beta and clarified in the text that this is an unnormalised rate matrix.

Line 44: The author should also make it explicit here that Q describes the rate from the row to the column (not column to row as some fields prefer).

Added “from row to column”

Lines 36-44: the author describes one commonly used parameterisation of the covarion model. But the BEAST 2 covarion implementation allows for three possible parameterisations, only one of which is described here. I suggest that all three parameterisations are acknowledged and contrasted, even if only one is being used in the rest of the paper. This will also be helpful for users who may be confused about this setting. The parameterisations are shown in the comments section at the top of the class below, and I have also copied them here for convenience:

https://github.com/CompEvol/beast2/blob/master/src/beast/base/evolution/substitutionmodel/BinaryCovarion.java

```
/**
 * <p/>
 * a    the rate of the slow rate class
 * 1    the rate of the fast rate class
 * p0    the equilibrium frequency of zero states
 * p1    1 - p0, the equilibrium frequency of one states
 * f0    the equilibrium frequency of slow rate class
 * f1    1 - f0, the equilibrium frequency of fast rate class
 * s, s1, s2 the rate of switching
 * <p/>
 * then the (unnormalized) instantaneous rate matrix (unnormalized Q) should be (depending
on mode)
 * <p/>
 *
 * mode = BEAST -- using classic BEAST implementation, reversible iff hfrequencies = (0.5,
0.5)
 * FLAGS: reversible = false, TSParameterisation = false
 *
 * [ -(a*p1)-s ,   a*p1    ,    s   ,   0   ]
 * [   a*p0    , -(a*p0)-s ,    0   ,   s   ]
 * [    s      ,     0     ,  -p1-s ,  p1   ]
```

```
* [    0      ,     s     ,     p0  , -p0-s ]
*
* equilibrium frequencies
* [ p0 * f0, p1, * f0, p0 * f1, p1, * f1 ]
*
* mode = REVERSIBLE -- brings in hfrequencies in rate matrix
* reversible = true, TSParameterisation = false
* [ - , a , s , 0 ]
* [ a , - , 0 , s ]
* [ s , 0 , - , 1 ]
* [ 0 , s , 1 , - ]
*
* which with frequencies becomes
*
* [ -(a*p1*f0)-s*f0 ,   a*p1*f0       ,    s*f0      ,   0         ]
* [   a*p0*f0       , -(a*p0*f0)-s*f0 ,    0         ,   s*f0      ]
* [    s*f1         ,     0           , -p1*f1-s*f1 ,  p1*f1      ]
* [    0            ,     s*f1        ,    p0*f1     , -p0*f1-s*f1 ]
*
* equilibrium frequencies
* [ p0 * f0, p1, * f0, p0 * f1, p1, * f1 ]
*
* mode = TUFFLEYSTEEL uses alternative parameterisation: hfrequencies is ignored, and
switch parameter is set to dimension = 2
* [ -(a*p1)-s1 ,   a*p1      ,    s1   ,   0    ]
* [   a*p0     , -(a*p0)-s1 ,    0    ,   s1   ]
* [    s2      ,     0       , -p1-s2 ,  p1    ]
* [    0       ,     s2      ,    p0   , -p0-s2 ]
*
* equilibrium frequencies
* [ p0 * s2/(s1+s2), p1, * s2/(s1+s2), p0 * s1/(s1+s2), p1, * s1/(s1+s2) ]
*
*
* Note: to use Tuffley & Steel's methods, set a = 0.
*/
```

I have added a paragraph to explain the three set-ups.

Jordan Douglas

Journal Requirements:

1) We ask that a manuscript source file is provided at Revision. Please upload your manuscript file as a .doc, .docx, .rtf or .tex. If you are providing a .tex file, please upload it under the item type u2018LaTeX Source Fileu2019 and leave your .pdf version as the item type u2018Manuscriptu2019.

2) Please provide an Author Summary. This should appear in your manuscript between the Abstract (if applicable) and the Introduction, and should be 150-200 words long. The aim should be to make your findings accessible to a wide audience that includes both scientists and non-scientists. Sample summaries can be found on our website under Submission

Guidelines:
https://journals.plos.org/ploscompbiol/s/submission-guidelines#loc-parts-of-a-submission

3) Please upload all main figures as separate Figure files in .tif or .eps format. For more information about how to convert and format your figure files please see our guidelines: https://journals.plos.org/ploscompbiol/s/figures

Reviewers' comments:

Reviewer's Responses to Questions

Comments to the Authors:
Please note here if the review is uploaded as an attachment.

Reviewer #1: This paper provides a much needed sanity check of the established "standard" methodology for Bayesian phylogenetics applied to linguistics, paying particular attention to the effects ascertainment bias on model inferences, as well as to the overall validity of the covarion model on linguistic data.

It provides a well motivated simulation study, seemingly one of the first studies specifically investigating the validity of models as they relate specifically to language/lexical evolution (though previous studies on the effects of lexical borrowing on tree topologies have been carried out e.g. Greenhill et al. 2009). The introduction presents a clear and fairly accessible summary of the key parts of the standard methodology which are later dissected in the paper, as well as a nice bibliography of phylolinguistic papers from the last 15 years or so.

The methods and discussion section is detailed, and comprehensive. A descriptive overview of the methods used to validate phylogenetic models is provided, and is easy to follow. It sets a standard for further model testing in phylolinguistics, even if the validation methodology has been presented elsewhere, and serves as a useful reference for computational historical linguists.

You have shown convincingly that the established model itself is identifiable so long as partitions are weighted by number of meanings not sites, and of course this is not difficult for future analyses to take into account. The discussion on the inadequacy of the binary covarion substitution model is interesting and articulates reasons why this may be the case. While the claim that the covarion is not a comprehensive or causally realistic model for "all aspects of lexical evolution" is likely uncontroversial, it's informative to have a more detailed explanation of the ways in which the model falls short. A short overview of current or future alternatives is included.

The code is all available and clear to read, as well as the BEAST2 .xmls for more practical instruction of how to implement the recommendations regarding partition weighting.

It is clear that bayesian phylogenetic analysis has become a indispensable tool for linguists of the modern era, and without sufficient validation of the methodology the field opens itself up to drawing conclusions based on model misspecification. This is particularly the case since the users of these methods often come from less technical backgrounds, and are more likely

to use the default BEAST2 settings for example.

I enjoyed reading the paper, and have a few minor suggestions for improvement:

1. including a diagram of the covarion model could help to illustrate it in a more accessible way, though this is already done e.g. in the cited Hoffman et al. paper and elsewhere
This would only duplicate the information already shown in the Q matrix, so I decided against it.

2. In your SBC: simulating trees of 30 taxa according to a yule process seems reasonable to me, since you then test the adequacy according to a best-case scenario and show that under these ideal conditions, BEAST2's default partition weighting results in failed coverage and RUV checks. However, from a practical perspective, I would be interested to know how you expect the bias to scale with number of cognates and meanings, number of taxa, or tree calibrations.
Added the sentence: “Since the partition rates in the SBC test are drawn from distributions similar to those found in empirical analyses, problems should not necessarily increase on larger datasets than those tested.”

3. I would appreciate a discussion of the impact of these findings on past research: particularly, how serious are the consequences of the ascertainment bias misspecification, are all trees with meaning rate heterogeneity/partitioning inferred in the literature of the past 10 years slightly longer than they should be etc.

I have expanded figure S5 to show not only clock rate, but also the other parameters, which are unaffected. I have added a new paragraph discussing consequences for previous studies (which in short are confined to the clock rate and partition rates).

4. The conclusion's point alluding to the covarion's “shortcomings” seem less severe than in both the abstract and in the previous discussion section “suggesting model misspecification”. I find myself wondering how severe it actually is, given its framing in the conclusion.

Changed shortcomings to misspecification.

5. there are a few instances where “modelling” is spelt inconsistently, the sentence at lines 211-212 seems basically a duplicate of the one before, quotation marks are typographically wrong e.g. line 90 ”look like” instead of “look like”

Changed to consistent “modelling”. The duplicate sentence is removed. Fixed the quotation marks issue.

Reviewer #2: This manuscript is a welcome and timely contribution to the methodology of phylogenetic inference as applied to linguistics, i.e. phylolinguistics. Its primary aim is to assess the validity and adequacy of phylogenetic models for languages using simulation-based tests. The study effectively demonstrates the limitations of current models and identifies key areas requiring further attention. The relevant literature is thoroughly and appropriately cited, and the methods employed are well-suited to address the research

questions. As such, this work represents a valuable contribution to the field and should be published, provided the author addresses the points outlined below. Given that some revisions and additions may be necessary, I recommend "major revisions". However, I am confident that, once my suggestions are addressed, the manuscript will be ready for publication.

Major suggestions

The title is too broad. It should explicitly state which specific aspects of validity and adequacy in phylolinguistic analyses are tested in the article.

I thought about changing it to: *"Testing the validity and adequacy of linguistic phylogenetic analyses through simulation-based calibration and posterior predictive simulations"* but it's overly convoluted. I've left the original title in place but if it is considered an essential revision it can be changed.

If this article is aimed at linguists working on phylogenetic inference, it should be made more accessible. First, the distinction between validity and adequacy should be clarified with more details. What would it mean to have a valid but inadequate model, or conversely an invalid but adequate model? The author should give concrete recommandations or warnings about the consequences of the available methodological choices. The discussion and the conclusion are probably too short.

If this article is intended for linguists working on phylogenetic inference, it should be made more accessible. The distinction between validity and adequacy requires further clarification. What would it mean for a model to be valid but inadequate, or invalid but adequate?

Added: Beyond validating the implementation of phylolinguistic analyses, there is also the question of model adequacy. Whereas validation ensures correctness of the computation in the inference machinery, adequacy refers to the how well the model represents the true process being modelled. It is possible that an analysis can be valid, in that the correct parameter estimates for a given model and dataset are estimated, but nevertheless inadequate if the model is mismatched to the underlying process.

The author should provide concrete recommendations or warnings about the implications of methodological choices. The discussion and conclusion sections are too brief and should be expanded to address these points more thoroughly.

Added two sections:

Weighting partitions by the number of meanings rather than the number of cognates should be the recommended practice in phylolinguistics going forward. An explicit weighted dirichlet distribution prior on the partition rates is also recommended, because an explicit prior distribution is lacking in the current BEAST2 default set-up.

Researchers performing phylolinguistic analyses should be aware that treating each cognate set as independent, and the inability of current models to account for semantic shift, may distort results.

The rationale behind the Rank Uniformity Validation procedure should be explained in

conceptual terms to aid understanding.

Added the following section:

A second test is Rank Uniformity Validation (RUV). It has been shown that any particular draw of parameter values from the prior distribution is also a draw from the posterior distribution conditional on data generated by that same prior draw \cite{cook2006validation}. In other words, in SBC, the true value used to generate the data (the prior draw), and the parameter estimates produced when that data is analysed (the corresponding posterior draws), should come from the same distribution provided the analysis is correctly implemented. It follows that when the posterior draws are ordered by rank, the true value has an equal probability of falling between any two of the posterior draws. The rank of the true value within the corresponding posterior draws should follow a uniform distribution (\nameref{Fig. S1}).

The terms "aligned" and "alignment" are misleading in a linguistic context. For example, on p. 5: "Validation simulation study 1, in which the simulated cognate alignments..." In phylolinguistics, "cognate alignment" typically refers to the alignment of sounds between word forms. While "alignment" and "sites" are standard terms in molecular phylogenetics, they do not directly apply to morphological or linguistic phylogenies. The author should rephrase these terms to avoid confusion.

Changed alignment throughout to “simulated data” or similar. Sites changed to “cognates”

Partitioning characters according to the number of cognate sets by meaning is common in phylolinguistic analyses. However, this raises the point of potential double dipping: we look at our particular data (cognate classes) to build a model and then use that model to analyse the same data. We can expect such a model to fit the data comparatively well, which is indeed the result quoted by the author. How much we should be concerned by this is beyond my expertise, but I know some colleagues are worried about this. It may be useful to discuss this point.

Partitioning characters based on the number of cognate sets by meaning is common in phylolinguistic analyses. However, this raises the issue of potential double-dipping: researchers use the same data (cognate classes) to both build a model and test it. Such models may naturally fit the data well, as results quoted by the author suggest. While the extent of this concern is beyond my expertise, I know that some colleagues are apprehensive about this practice. A discussion of this point would be valuable.

In Bayesian phylogenetics it is common practice to use some sort of quick analysis of the dataset to determine the partitioning scheme, for example in the programs partitionfinder or clockstar. Double dipping in my understanding means using the data to come up with a prior distribution. This is not being done in this case, the partition rates are all given the same prior distribution. It’s not the case for example that the partitions with more cognate-sets per meaning are given a prior with a higher median rate.

The author notes that empirical analyses rarely encounter all-absent cognate classes and

typically include dummy columns in the character matrix to correct for missing data. However, the status of all-absent cognates in the simulation-based calibration study remains unclear. Were these generated because some cognate classes disappeared before reaching the tips during simulated evolution? Are these simply ascertainment bias correction columns? For Simulation Study 3, the partition rates were re-weighted, but the weighting criterion is not specified. It should also be made clear from the outset that this section focuses on testing the ascertainment bias correction procedure.

Added the following section:

Simulated data differs from empirical data because all-absent cognates are present. All-absent cognates in the simulations primarily result when simulations begin in the absent state at the root, and no transitions to the present state occur along the tree. When the transition rate from absent to present is low (either due to a slow clock rate or a low stable frequency of the present state), a correspondingly larger proportion of the 10,000 simulated cognate sets are all-absent. Three versions of the SBC were performed to test the ascertainment bias correction, based on different treatments of all-absent cognate classes and the weighting of the 3 partition rates.

Figure 4 is difficult to interpret: What does the red staircase-like line represent? What does the purple cloud around the line signify?

Changed the figure caption:

The stepped nature of the graph is because of binning of nodes with a range of posterior probabilities. The purple area represents 90 \% confidence bands.

Table 2 is also difficult to interpret: What is the denominator used to compute the Bayes factor? The large value in the Bayes factor column suggests that the "reweighted by number of cognates" model is the best, but this is not the case. Is there an error in the table?

Changed the table to Bayes factors vs best, i.e. worse fitting models now have values below 1

Minor comments

On p. 1, the author equates lexemes with word forms, but strictly speaking, a lexeme is an abstract unit defined as a set of word forms that share a lexical meaning and morphosyntactic properties, forming an inflectional paradigm. In practice, linguists record a representative form for the lexeme used to express a concept. A reference to literature describing the standard workflow (e.g., works by Greenhill et al. or List et al.) would be helpful.

added:

Lexemes, specifically the canonical word form, are collected for each language in a given set of meanings

Also included the greenhill reference.

On pp. 1–2: "Each cognate is treated as an independent character" should be clarified. More precisely, for binary-state characters, characters are pairs consisting of a concept and a cognate set.

Changed to:

Each cognate is treated as a character with two states: absent (0) and present (1), and treated independently from other cognates in the same meaning

On p. 2: "Cognates that are present in all languages, although invariant, can and are observed." While true, the author could add that this occurs because linguists typically use a standard list of concepts. In some language families, after data collection, researchers may observe no variation for a concept, i.e. a single cognate set exists. However, some studies choose to remove invariant characters, e.g. Goldstein, David. 2024. Divergence-time estimation in Indo-European. Diachronica 41(1). 1–45. https://doi.org/10.1075/dia.22031.gol.

Changed to:

Cognates that are present in all languages, although invariant, can and are observed: because phylolinguistics draws upon a fixed set of meanings, occassionally all lexemes in a particular meaning come from the same cognate set.

On p. 5: "I extracted the subtree of these languages from the summary tree in [3], and fixed this during the analysis." It is unclear what was fixed. The author should specify this.

added:

This tree was fixed during the analysis, and estimated the values of the binary covarion and relaxed clock parameters in BEAST2.

In Figures 2.jpg and S3 Fig.jpg, the blue and red colors are not visible in some panels because small intervals are obscured by black data points and the diagonal black line. To improve readability, the diagonal could be drawn with gray dots, and the data points could be represented as empty circles with outlines.

I have decreased the size of the points and made the line gray dots.

Reproducibility

Instructions and comments for running the code are minimal (except for the simulation pipeline), and the project is difficult to navigate, making reproducibility challenging.

I have expanded and improved the readme with more explanations and a clearer structure.

The provided R code relies on `setwd(dirname(rstudioapi::getSourceEditorContext()$path))`, which depends on RStudio and is not listed in the requirements. A project-oriented workflow (e.g., rstats.wtf/projects) would be preferable.

Added R Studio to the requirements

While the BEAST XML configuration files are provided, running all analyses is time-consuming. The author should also include the resulting log and tree files, as well as marginal_likelihood.txt and other output files. Currently, only the code to generate these files is provided. An undocumented and hidden file (IndoIranic_modeltesting/pathsampling/.RData) was mistakenly included and should be removed.

log files are now included

To ensure long-term reproducibility, the author should include a version of the asr_functions.R file in the project, rather than reading it from GitHub. This would allow the code to run offline, even if GitHub becomes unavailable or the file is modified or relocated.

The functions are now in the project as suggested

Have the authors made all data and (if applicable) computational code underlying the findings in their manuscript fully available?
The PLOS Data policy requires authors to make all data and code underlying the findings described in their manuscript fully available without restriction, with rare exception (please refer to the Data Availability Statement in the manuscript PDF file). The data and code should be provided as part of the manuscript or its supporting information, or deposited to a public repository. For example, in addition to summary statistics, the data points behind means, medians and variance measures should be available. If there are restrictions on publicly sharing data or code —e.g. participant privacy or use of data from a third party—those must be specified.

Reviewer #1: Yes

Reviewer #2: Yes

PLOS authors have the option to publish the peer review history of their article (what does this mean?). If published, this will include your full peer review and any attached files.

If you choose "no", your identity will remain anonymous but your review may still be made public.

Do you want your identity to be public for this peer review? For information about this choice, including consent withdrawal, please see our Privacy Policy.

Reviewer #1: No

Reviewer #2: **Yes:** Thomas Pellard

Reviewer 3

The author proposes a statistical test of the standard phylolinguistics framework, using SBC (Simulation Based Calibration). This topic is timely and well suited to PLOS Computational Biology. I believe it will be of interest to the scientific community, and hope it will eventually be published.
For context, I am a statistician with long experience in phylolinguistics. Overall, I found the article to be well written. I have a number of comments and requests for clarification. My assumption is that these will relatively straightforward to implement. I am nonetheless recommending a major revision because of point 1 below.

1 Major comments

1. The code and data should be made available on a public repository such as github. I would have liked to check the code and reproduce the figures, but was unable to do so. When the paper is revised, I would like to be able to verify the reproducibility.
The code was available and is again for the revision.

2. The paper discusses ascertainment bias and partition rate re-weighting. It would be a good idea to explain these two notions in more detail, including with some technical discussion of they are implemented. In fact, to the uninformed reader, it might not be clear whether rate re-weighting is a technique for ascertainment bias or is a separate issue.
Added paragraphs explaining these two issues and how they might interact.

3. Linked to the previous comment: in the description of the 6 models, specify what was done for ascertainment bias correction.

Edited to show that models 2 and 3 had the ascertainment bias correction applied.

4. There is no discussion of checking the convergence and mixing of the MCMC algorithms.

To confirm that the MCMC chains had run sufficiently long, I calculated effective sample size (ESS) scores for each free parameter in each simulation replicate using the R package sns \cite{Alireza2016sns}. ESS scores were over 200 for all parameters in all simulation replicates.

5. I am confused by the claim line 167 that rate parameters, tree height, and tree length are well-calibrated. For these quantities to be well estimated, we typically need some constraints on internal node ages, but these constraints were never mentioned. (Without constraints, you can get the same likelihood by multiplying

all branch lengths by a constant, and multiplying the rates by the same constant).

I fixed the clock rate. This was in figure 1 but is now also included in the text.

6. Various metrics are designed to search for mis-specification, but they are never described, nor even listed apart from the labels of Figure 6. Please describe these and give intuition of why they are interesting quantities.

The metrics were already described in the methods. I have added an introductory sentence to describe the main aims of these metrics.
For comparison of simulated and empirical datasets I calculated 8 metrics, designed to capture key aspects of language divergence, the composition of languages (in terms of cognates), and the distribution of cognates across languages. For languages I calculated the maximum, minimum and mean pairwise distance between languages, mean proportion of present cognates and variance in the number of present cognates. For cognates I calculated the number of prevalent cognates (prevalent defined as greater than 80\% of the language sample), number of singletons (cognates present in one language, autapomorphies) and variance in cognate prevalence.

7. Various mis-specifications are listed at the end of the final section. But do these mis-specifications matter? Do we have reason to believe that missing these misspecifications lead to poor inference of the quantities of interest?

Model inadequacy, as shown by posterior predictive simulations, raises the question of how the estimation of the parameters of interest is affected. In a phylolinguistic analysis the parameters of interest are usually node ages and tree topology. Node age estimates could conceivably be affected by semantic shift, for example if differences between closely related languages are inflated more relative to the differences between distantly languages. Node age estimates may also be affected by the non-independent evolution of cognates. This is demonstrated by previous implementations of a multistate model, which produced younger (arguably unrealistically young) node age estimates when compared to the binary covarion model. Future work to explore how the inadequacy of the covarion model affects node age estimates is needed.

2 Minor comments

8. line 47, the term ”rates of lexical replacement” is ambiguous: it is easy to interpret it as the rate at which a cognacy class changes in the multistate model. I would suggest the more neutral ”transition rate”.

One way of modelling this is to apply different rate multipliers to each meaning partition. These rates are hereafter referred to as "partition rates", but are often called "mutation rates" within the beast2 software

9. If I understand correctly line 105, the simulated data sets comprise close to 30 000 cognacy classes. This is about 10 times larger than typical analyses in phylolinguistics, and should be commented upon.
Added:
The large number of simulated cognates was necessary due to the large proportion of all-absent cognates in the simulated data.

10. In Figure 1, I don’t understand what ”i ∈ 3” and ”i ∈ 58” mean.
Added explanation to the figure caption

11. I got confused by the numbering of the studies: when you refer e.g. to ”analysis 3”, it isn’t clear if this is synthetic data analysis 3 or empirical data analysis 3. Maybe rename the analyses.

Changed to “mirroring the results found in the SBC tests” to avoid this ambiguity.

12. p. 5: note that if there were missing data, that would change the ascertainment: in practice, data which are absent or missing at all leaves are removed (even though the cognate may have in fact been present at a leaf where it is recorded as missing). This is discussed e.g. by Ryder and Nicholls (2011), for the less-used Stochastic Dollo model.

Added:

Note that for empirical datasets with missing data, the ascertainment bias correction is slightly modified, since the probability of an all-absent increases as the proportion of missing data increases \cite{chang2015ancestry}.

13. The interpretation of the SBC plots could go into more detail. For instance, remind us how to interpret the profiles of the plots you obtain.

Captions for figures 2 and 3 have been expanded, and figs S1-2 referenced therein.

14. Table 2: this is the log-marginal likelihood. Also, these are Bayes factors against each model. At first glance, I interpreted the 85.7 BF as meaning that the first line was the favoured model.

Changed the table to Bayes factors vs best, i.e. worse fitting models have values below 1. Included log in the column title.

15. I don’t understand the statement l. 215 that all cognate sets are eventually lost. Do you mean that they eventually become impossible to identify because of phonological changes? That is a separate issue.

Changed to:

Arguably, the stable frequency of the present state for any given cognate is even more extreme than that estimated by the analysis, and is in fact zero. When cognates are truly lost (rather than undergoing semantic shift) this process is not reversible. Over very long timescales any given cognate will eventually be lost, although many can persist for thousands of years.
